# Supplementary material for: The Mechanisms Underlying Salt Resistance Mediated by Exogenous Application of 24-Epibrassinolide in Peanut
Source: Int J Mol Sci. 2022 Jun 7;23(12):6376. doi: 10.3390/ijms23126376 (PMC9224412; doi:10.3390/ijms23126376)
Supplement: Supplementary file 1 [file ijms-23-06376-s001.zip › ijms-1703179-supplementary.pdf]

Supplements

1. Supplementary results

1.1. Effects of NaCl stress on the growth and physiological indexes of peanut seedlings

The growth of peanut seedlings was decreased with increasing concentration of NaCl (Fig 1A). Compared with control, 50 mM NaCl treatment significantly decreased the height of main stem (Fig 1B), while there was no significant difference in root length and fresh weight (Fig 1C). Compared with control, 100 mM, 150 mM and 200 mM NaCl treatments significantly reduced the main stem height and fresh weight of peanut seedlings (Fig 1B and 1D), Compared with 100 mM NaCl, the seedling leaves under 150 mM and 200 mM NaCl treatments withered obviously. Compared with control, 150 mM and 200 mM NaCl treatment significantly increased the content of MDA in seedling leaves (Fig.1E). Finally, 150 mM NaCl was selected as salt stress concentration.

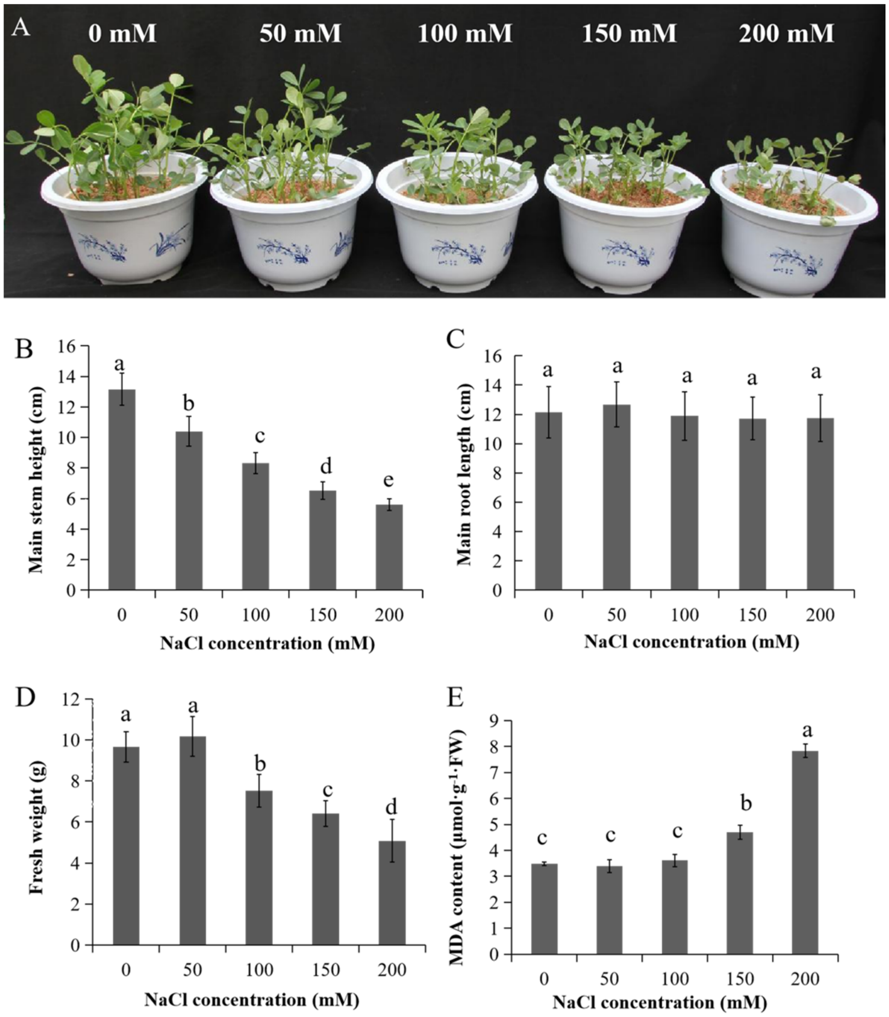

Figure S1. Effects of NaCl stress concentrations on the growth and physiological indexes of peanut seedlings.

(A) Seedling morphology; (B) main stem height; (C) main root length; (D) seedling fresh weight; (E) MDA content. Different lowercase letters indicate significant differences between treatments ($P < 0.05$).

1.2. Exogenous EBL Treatment Improved the Salt Tolerance of Peanut Seedlings

Peanut seedlings grown under 150 mM NaCl treatment were sprayed for 6 d with 0 μM, 0.001 μM, 0.01 μM, 0.1 μM and 1 μM EBL, respectively. As shown in Fig. 2, EBL had a dose-dependent manner on the growth of peanut seedlings under 150 mM NaCl treatment. With the increase of EBL concentration, main stem height and fresh weight were increased first and then decreased. Exogenous Spraying 0.1 μM EBL effectively alleviated the wilting of seedling leaves, increased the main stem height and fresh weight, and reduced the MDA content of peanut seedlings (Fig. 2E). Therefore, 0.1 μM EBL was selected for further experiments.

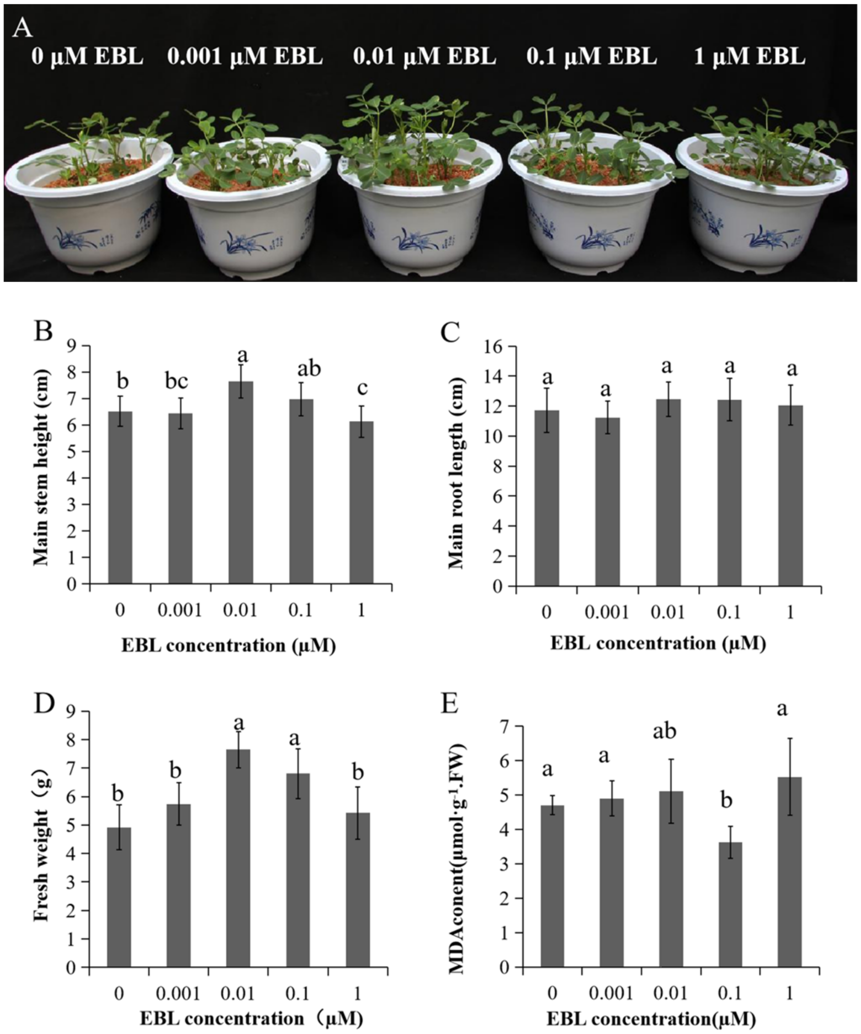

Figure S2. Effects of EBL on the growth and MDA content of peanut seedlings under NaCl stress. (A) Seedling morphology; (B) main stem height; (C) main root length; (D) seedling fresh weight; (E) MDA content.

2. Supplementary Methods

2.1. Screening of NaCl stress concentration

Peanut seedlings of 12 d were selected and divided into five groups for treatments, 0 mM (CK), 50 mM, 100 mM, 150 mM and 200 mM NaCl. The NaCl dissolved in Hoagland's nutrient

solution. After 6 d NaCl treatments, plant fresh weight, the main stem height, root length and MDA content were determined.

2.2. EBL concentration screening

Peanut seedlings were selected and divided into five groups for treatments, Exogenous spraying of 0 μM EBL, 0.001 μM EBL, 0.01 μM EBL, 0.1 μM EBL and 1 μM EBL under 150 mM NaCl was carried out. The stock solution (1 mM) of EBL (Sigma Chemicals, USA) was prepared by dissolving EBL in ethanol and was stored at -20℃. The final concentration of EBL was prepared using double distilled water. After 6 d treatment, plant fresh and dry weight, the main stem height, root length and MDA content were determined.
